# Self-reported pain on a digital workplace assessment: identifying the score that flags desk-based workers for earlier occupational health review

**DOI:** 10.3389/fdgth.2026.1919657

**Published:** 2026-08-18

**Authors:** Tsz Ling Nicola Tik

**Affiliations:** Vitrue Health, London, United Kingdom

**Keywords:** digital health, musculoskeletal disorders, occupational health, pain measurement, presenteeism, work disability prevention

## Abstract

**Introduction:**

Musculoskeletal pain is common among desk-based workers and reduces productivity, increases healthcare use, and contributes to work disability. Digital workplace platforms now record self-reported pain at scale, offering a way to use a single score to flag workers for earlier review, yet evidence on the lowest score at which pain is consistently associated with functional impact across multiple domains is lacking. This study aimed to identify that point.

**Methods:**

Retrospective cross-sectional analysis of de-identified data from 11,529 working adults reporting pain who completed digital desk-based musculoskeletal assessments between January and November 2025. Pain was self-reported on an 11-point scale (0–10) with defined verbal descriptors at each level (a descriptive pain scale). Five functional domains were examined: productivity impairment, healthcare-seeking, mental health impact, social participation, and work absenteeism. Logistic regression estimated odds ratios (ORs) with 95% confidence intervals, with a score of 1 as reference, adjusted for age and sex and with cluster-robust standard errors accounting for clustering of workers within employing organisations (116 organisations); generalised estimating equations were used as confirmation.

**Results:**

A pain score of 4 was the lowest level at which four of the five domains, productivity, healthcare-seeking, mental health, and social participation, were significantly associated with pain after adjustment and clustering (all *p* < 0.001; ORs: 3.5–5.3). Absenteeism showed an association of similar magnitude (OR: 2.0) but with a confidence interval that included once organisational clustering was accounted for, consistent with it being the rarest and most downstream outcome. Associations strengthened progressively at higher scores.

**Conclusion:**

A self-reported pain score of 4 was the lowest level at which functional impact was consistently associated with pain across four independent domains, robust to age, sex, and organisational clustering. These cross-sectional associations may inform use of a score of 4 on this anchored scale to prompt earlier review; they do not establish that intervening changes outcomes, and the threshold is specific to this anchored scale. Prospective studies are required.

## Introduction

Musculoskeletal (MSK) conditions are the leading cause of years lived with disability globally, affecting an estimated 1.71 billion people and accounting for the largest share of rehabilitation need across 134 of 204 countries analysed in the Global Burden of Disease Study (1, 2). In working populations this burden takes a distinctive and costly form. Among office and desk-based workers, a group characterised by prolonged static postures, sedentary behaviour, and sustained computer use, the prevalence of work-related MSK pain is consistently high, with systematic reviews reporting rates from 50% to over 80% across the neck, shoulder, and lower back (3, 4). MSK conditions are the leading reason for sickness absence and work disability in the United Kingdom, and their consequences extend well beyond physical symptoms: persistent pain in working adults is associated with reduced productivity, poorer mental health, social withdrawal, and increased healthcare use, all of which carry substantial costs to individuals, employers, and health systems (5, 6).

The economic burden is considerable. In the United Kingdom, presenteeism (reduced productivity from working while unwell) is estimated to cost employers around £103 billion annually, with MSK conditions among the leading drivers (6). Musculoskeletal disorders account for a global welfare loss exceeding US$2 trillion, equivalent to 1.41% of global GDP in 2021 (7). For employers, the costs of MSK-related presenteeism consistently exceed those of absenteeism, yet workplace health systems are more often organised around sickness absence than around the earlier, more prevalent problem of functional impairment at work (8).

Despite the scale of this burden, occupational health practitioners lack empirically grounded thresholds for deciding when pain intensity warrants active review. Self-reported numerical pain scales are widely used in clinical and workplace assessment and are increasingly embedded in digital platforms that collect pain data at scale. Established occupational instruments such as the Standardised Nordic Musculoskeletal Questionnaire capture the presence, site, and work interference of symptoms, and are validated for epidemiological surveillance rather than for grading the functional consequences of a given pain intensity. Digital and sensor-based methods for assessing musculoskeletal risk and postural stress are also developing rapidly, extending traditional observational approaches (9–11). Broader reviews of ergonomic assessment methods for work-related musculoskeletal disorders provide context for how exposure and symptoms are measured in occupational settings (12–14).

The functional consequences of pain in working adults span several conceptually distinct dimensions recognised across occupational medicine, ergonomics, and occupational psychology. We grouped these into five domains: productivity impairment, the capacity to work effectively, encompassing presenteeism (15, 16); healthcare-seeking behaviour, the use of professional care (17, 18); mental health impact, the psychological burden of pain (19, 20); social participation, interference with social and daily roles (21, 22); and work absenteeism, time lost from work (23, 24). Existing threshold analyses are largely derived from clinical populations and single outcomes, such as analgesic response or cancer pain classification, and rarely consider these occupational dimensions together. If pain intensity and functional impact are not linearly related, and there is a point at which consequences become consistently significant across multiple independent domains at once, this would have direct implications for when occupational health review should begin.

Using data from a digital workplace assessment platform, this study examined the relationship between self-reported pain intensity and functional impact across these five domains. The objective was to determine whether there is a pain score at which the odds of functional impact become consistently and significantly elevated across domains simultaneously, and thus whether a single routinely collected score could serve as an empirically grounded signal for earlier occupational health review.

## Methods

### Study design and data source

This retrospective cross-sectional study analysed de-identified data from working adults who completed desk-based musculoskeletal pain assessments through the VIDA digital workplace wellness platform (Vitrue Health, London, UK) between 1 January and 24 November 2025. The platform delivers standardised assessments to employees of employer clients participating in workplace wellness programmes. The analysis was conducted by the author, an employee of Vitrue Health, using a fully de-identified extract from the platform database from which all direct identifiers had been removed; the author did not access identifiable participant data at any point. The analysis used a de-identified extract from the platform database from which all direct identifiers had been removed; each record carried only a coded user ID that could not be linked to an individual by the author, who did not access identifiable participant data at any point. As a service evaluation of routinely collected, de-identified data with no modification to standard care pathways, NHS Research Ethics Committee review was not required under UK Health Research Authority guidance.

### Participants

Working adults aged 18 years and over who completed a desk-based musculoskeletal pain assessment during the study period were eligible. The analytic sample comprised 11,529 assessments reporting any pain (score 1–10) from employees of active employer clients. Assessments recording no pain (score 0) were excluded, consistent with the aim of characterising the relationship between pain intensity and functional impact among those reporting pain. The mean age was 38.4 years (SD: 10.9); 58.8% of participants were recorded as female, 35.1% as male, and the remainder as another category or not recorded.

### Pain intensity measurement

Pain intensity was measured on an 11-point scale (0 = no pain, 10 = worst imaginable pain) in which each level carried a defined verbal descriptor. This format, a descriptive pain scale, pairs each point on the 0–10 scale with a functional anchor statement describing the experience of pain at that level, and has the advantage that all respondents rate against the same explicit functional definitions rather than interpreting an unlabelled number individually. The platform's descriptors were adapted from the descriptive pain scale tradition, drawing on several publicly circulated versions of this scale type rather than reproducing a single named instrument; a comparable publicly available example is the descriptive pain scale published as a clinical resource by Oregon Health & Science University (25). The descriptor for a level appeared when the respondent hovered over the corresponding number and remained displayed alongside the selected score, so that the functional definition of the chosen level (for example, a score of 4 was defined as “distressing: constantly aware of my pain but can continue most activities”) was visible at the point of selection. This provided a consistent functional reference for each score across the sample, supporting comparability of ratings between respondents. The full set of descriptors as presented on the platform is provided in Supplementary Table S1. Whether each participant read the descriptor before selecting their score was not recorded, and the descriptors have not been formally validated as a standalone instrument; these points are considered in the limitations.

### Functional impact domains

Five functional impact domains were derived from self-report items administered at the time of pain assessment; all referred to the participant's pain overall rather than to individual body regions. Three domains (productivity impairment, mental health impact, and social participation) were derived from a single optional multi-select question, “What impact has your pain had on you?”, which offered seven options: anxiety, stress or mood changes; reduced work productivity; sleep disruption; interference with social life and daily tasks; difficulty concentrating; other; and “my pain doesn't really impact me.” Respondents could select any number of options. Selecting “my pain doesn't really impact me” corresponded to no impact endorsed, coded as absent on all three domains; the “other” option was not mapped to any domain and was not analysed. The full question and option-to-domain mapping are given in Supplementary Table S2. The remaining two domains (healthcare-seeking and work absenteeism) were derived from separate single-item questions described below.

#### Productivity impairment

Coded present (1) if the respondent endorsed one or more of three options from the multi-select question, reduced work productivity, sleep disruption, or difficulty concentrating, and absent (0) otherwise. These three were grouped *a priori* because each represents a recognised pathway through which pain reduces work capacity: reduced work productivity captures direct output loss (presenteeism); sleep disruption captures impaired next-day cognitive and physical function, a documented mediator of pain-related work impairment; and difficulty concentrating captures reduced attentional performance during work. A composite was used because the study question concerned overall capacity to work rather than any single mechanism, and the three are expected to co-occur.

#### Mental health impact

Coded present (1) if the respondent endorsed “anxiety, stress or mood changes” on the multi-select question, otherwise absent (0). This reflects the psychological burden of pain recognised in the biopsychosocial model.

#### Social participation

Coded present (1) if the respondent endorsed “interference with social life and daily tasks” on the multi-select question, otherwise absent (0). Referred to throughout as social impact.

#### Healthcare-seeking behaviour

Derived from the single-item question “Have you needed to see a healthcare professional for your pain?” Responses were dichotomised: “Yes” coded present (1); “No”, “No, but I'm considering it”, and “Prefer not to say” coded absent (0), capturing confirmed healthcare contact only.

#### Work absenteeism

Derived from the single-item question “Have you ever had to take time off work because of your pain?”, dichotomised using the same conservative rule (“Yes” = present; all other responses = absent).

### Statistical analysis

For each pain score from 1 to 10, the proportion of participants endorsing each domain was calculated. To examine the dose-response relationship, binary logistic regression estimated ORs with 95% CIs for each domain at each pain score, with a score of 1 as the reference. ORs provide an interpretable measure of the magnitude of association at each pain level, and the formal reference category allows systematic identification of the pain score at which each domain first reaches significance. The primary analysis was unadjusted.

For each pain score from 1 to 10, the proportion of participants endorsing each domain was calculated, and binary logistic regression estimated odds ratios (ORs) with 95% confidence intervals (CIs) for each domain at each score, with a score of 1 as the reference category. Pain score was modelled as a categorical variable so that the score at which each domain first reached significance could be identified without assuming a linear dose-response. Models were adjusted for age (years, continuous) and sex (female as reference; male; and a combined category for participants recording another gender or preferring not to disclose), fitted by complete-case analysis on participants with a valid recorded age between 18 and 80 years and a recorded sex (*n* = 10,453; 9.3% excluded). Because workers were nested within employing organisations, and observations within an organisation may be correlated, all adjusted models used cluster-robust (sandwich) standard errors with the organisation as the clustering unit (116 organisations). As a confirmatory check, each model was refitted using generalised estimating equations (GEE) with an exchangeable working correlation structure and the organisation as the grouping variable; results were materially unchanged and are reported in Supplementary Table S3. Cluster-robust estimates are reported as primary throughout.

A secondary descriptive analysis examined the incremental percentage-point change between adjacent pain scores across the full range, to characterise the pattern of accumulation without presupposing where any threshold lies, and to contextualise the OR findings.

Analyses were conducted in Python 3.12 using pandas, NumPy, and statsmodels (logistic regression with cluster-robust covariance, and GEE). All 11,529 participants provided complete responses to the five outcome items. The author used Claude (Anthropic) to assist with manuscript language editing and writing development; generative AI was not used to generate scientific content, data, or analysis, is not listed as an author, and the author has verified all data, quotes, citations, and references and takes full responsibility for the integrity of the manuscript.

## Results

### Sample characteristics

The analytic sample comprised 11,529 assessments reporting any pain (score 1–10), drawn from employees of 118 employing organisations. The mean age was 38.4 years (SD: 10.9; median: 37, IQR: 30–46, Range: 18–78); 58.8% of participants were recorded as female, 35.1% as male, and the remainder as another category or not recorded. Baseline characteristics, including the distribution of pain score and each functional domain overall and by sex and age band, are presented in Table 1. Pain intensity was right-skewed, with the largest groups reporting a score of 3 (*n* = 2,712; 23.5%) or 4 (*n* = 2,330; 20.2%); pain of moderate or greater intensity (score 4 and above) was reported in 6,909 assessments (59.9%). Median pain score was 4 in both women and men. Overall domain prevalence was 41.3% for healthcare-seeking, 25.4% for social impact, 20.7% for mental health impact, 19.9% for productivity impairment, and 13.5% for absenteeism. Healthcare-seeking, mental health impact, and absenteeism were somewhat more frequently reported by women than men, whereas productivity impairment and social impact were similar by sex; a full sex-stratified analysis of the pain burden is reserved for a separate planned study.

**Table 1:** Baseline characteristics and prevalence of each functional domain, overall and by sex and age band (*N* = 11,529).

Characteristic	n	Mean age	Median NRS	Prod. %	HCP %	Mental %	Social %	Absence %
Overall	11,529	38.4	4	19.9	41.3	20.7	25.4	13.5
Female	6,782	38.4	4	19.6	44.3	22.3	25.6	14.3
Male	4,052	38.5	4	20	36.9	18.7	25.1	12.5
Age 18–29	2,513	25.4	4	24.9	30.9	22	22.6	11.6
Age 30–39	3,502	34.3	4	21.4	39.7	22.5	25	12.9
Age 40–49	2,521	44.1	4	17.6	49.4	21.5	27.6	15.5
Age 50–59	1,567	53.8	4	14.7	50	18.3	29.6	16.2
Age 60+	364	63	4	13.5	54.1	13.7	29.1	14.8

NRS, pain score on the 11-point descriptive scale; Prod., productivity impairment; HCP, healthcare-seeking. Percentages are the proportion within each row endorsing that domain. Sex categories shown are female and male; participants recording another category or not disclosing are included in the overall column.

### Dose-response relationships across five domains

**Figure 1:**
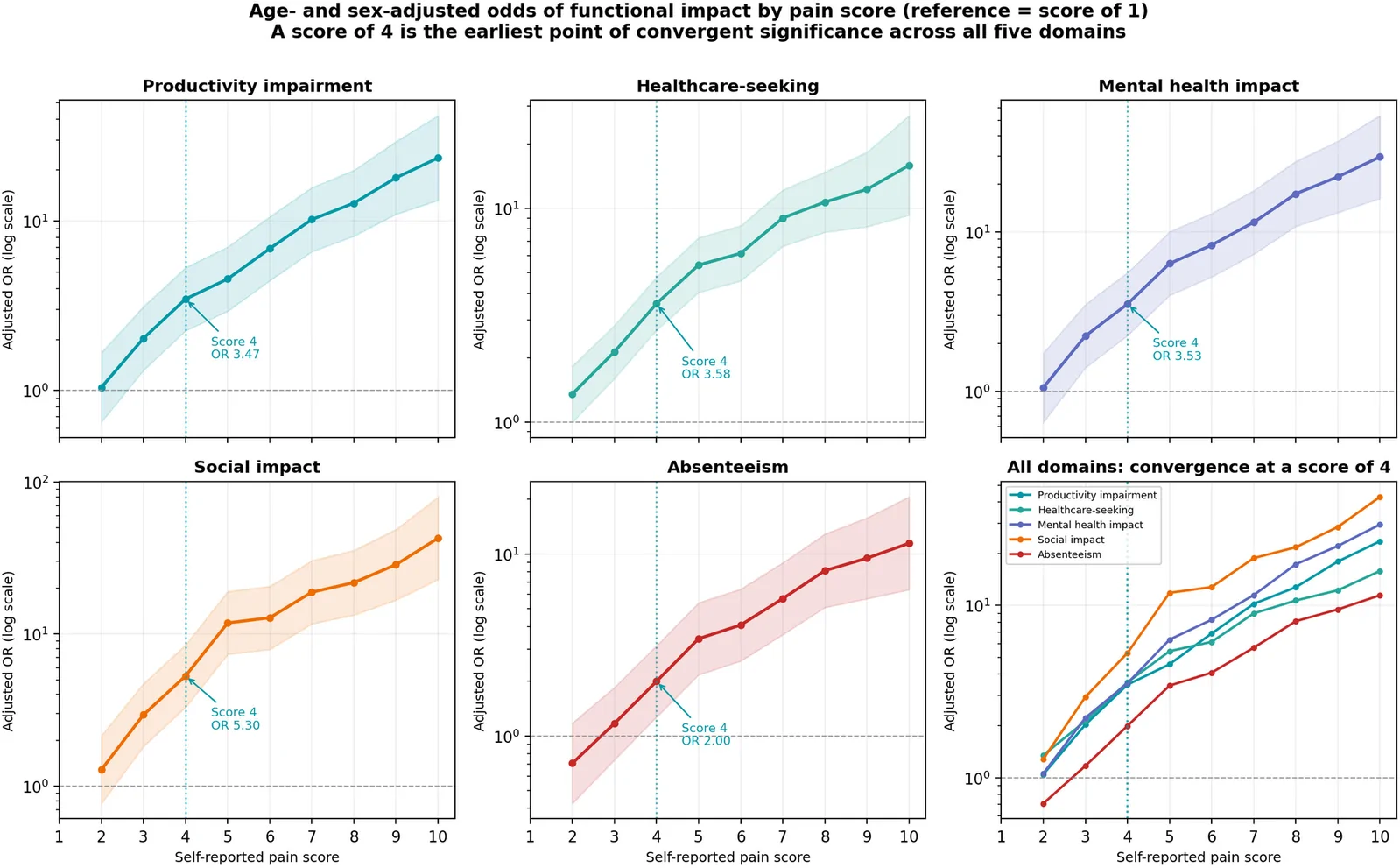
Age- and sex-adjusted odds of functional impact by pain score across five domains (reference = score of 1; cluster-robust models; complete-case *n* = 10,453). Each panel shows odds ratios with 95% confidence intervals on a logarithmic scale; the lower-right panel overlays all five domains. The dotted vertical line marks a score of 4, the lowest level at which the four multi-select and healthcare-seeking domains were simultaneously significant.

Across all five domains, the odds of functional impact increased progressively with pain intensity (Tables 1, 2, Figure 1). All ORs reported are adjusted for age and sex with cluster-robust standard errors accounting for organisational clustering. The pattern below a score of 4 was not uniform: healthcare-seeking and the three other multi-select domains reached significance at a score of 2 or 3 in some models, whereas absenteeism did not reach significance at any score below 4.

**Table 2:** Age- and sex-adjusted odds ratios (95% CI) for each functional domain by pain score, relative to a score of 1, with cluster-robust standard errors (organisation as clustering unit; *n* = 10,453; 116 organisations).

NRS	Productivity	Healthcare-seeking	Mental health	Social	Absenteeism
2	1.05 (0.61–1.78)	1.35 (0.83–2.21)	1.06 (0.71–1.58)	1.28 (0.78–2.12)	0.71 (0.41–1.23)
3	2.03 (1.20–3.45)**	2.13 (1.25–3.64)**	2.23 (1.40–3.55)***	2.94 (1.59–5.45)***	1.17 (0.61–2.24)
4	3.47 (1.94–6.20)***	3.58 (1.97–6.52)***	3.53 (2.36–5.28)***	5.30 (2.89–9.73)***	2.00 (0.96–4.15)
5	4.56 (2.35–8.84)***	5.42 (3.41–8.64)***	6.34 (4.16–9.66)***	11.84 (7.08–19.80)***	3.42 (1.69–6.96)***
6	6.88 (3.84–12.34)***	6.15 (3.89–9.74)***	8.27 (5.18–13.21)***	12.80 (7.98–20.52)***	4.07 (2.23–7.43)***
7	10.22 (5.98–17.45)***	9.00 (6.23–13.00)***	11.49 (7.36–17.95)***	18.85 (11.55–30.76)***	5.69 (3.54–9.14)***
8	12.77 (7.29–22.38)***	10.69 (7.25–15.74)***	17.35 (11.39–26.45)***	21.80 (12.79–37.17)***	8.10 (4.58–14.35)***
9	18.02 (12.44–26.09)***	12.26 (7.75–19.38)***	22.18 (12.15–40.49)***	28.61 (16.51–49.58)***	9.49 (6.90–13.05)***
10	23.61 (13.95–39.95)***	15.86 (9.67–26.02)***	29.54 (19.04–45.83)***	42.75 (21.82–83.74)***	11.46 (6.67–19.71)***

Values are odds ratios (95% confidence interval). Unmarked, non-significant. A score of 4 is the lowest level at which productivity, healthcare-seeking, mental health, and social impact are simultaneously significant. Absenteeism at a score of 4 has a similar point estimate (OR 2.00) but its interval includes 1 once organisational clustering is modelled. Confirmatory generalised estimating equation models are reported in Supplementary Table S3.

** *p* < 0.01.

*** *p* < 0.001.

### A pain score of 4 as the earliest point of convergent significance

A pain score of 4 was the lowest level at which four of the five domains were simultaneously and significantly associated with pain after adjustment for age and sex and with cluster-robust standard errors (Table 2). At a score of 4, the adjusted, clustered ORs were: productivity impairment 3.47 (95% CI: 1.94–6.20); healthcare-seeking 3.58 (95% CI: 1.97–6.52); mental health impact 3.53 (95% CI: 2.36–5.28); and social impact 5.30 (95% CI: 2.89–9.73); all four *p* < 0.001. Confirmatory GEE models gave materially identical estimates (Supplementary Table S3).

#### Absenteeism at a score of 4

Absenteeism showed an adjusted association of similar magnitude to the other domains (OR 2.00), but its confidence interval included once organisational clustering was accounted for (cluster-robust 95% CI: 0.96–4.15; GEE: 0.84–5.40), whereas it had been significant under standard errors that assumed independence (95% CI: 1.27–3.14). This attenuation reflects that absenteeism was the rarest outcome (13.5% overall) and that reported absence was concentrated within a subset of organisations, reducing the effective information once clustering was modelled. The point estimate nonetheless indicates roughly a doubling of the odds of absence at a score of 4 relative to a score of 1, and absenteeism became clearly significant at higher pain scores (for example, adjusted clustered OR: 3.42 at a score of 5). We therefore describe a score of 4 as the earliest point of convergent significance across the four domains that carry sufficient information to be estimated precisely, with absenteeism following the same directional pattern.

Age and sex were themselves associated with several outcomes in expected directions: older age was associated with greater healthcare-seeking but lower odds of productivity and mental health impact, and men had lower odds of healthcare-seeking than women. These covariate associations did not alter the score-of-4 convergence.

### Descriptive pattern of accumulation across the pain scale

Examination of incremental percentage-point changes between adjacent pain scores provides further context. From a score of 1–3, increases were modest across all domains. The score 3-to-4 transition produced consistent increases across all five domains simultaneously: healthcare-seeking rose by 11.2 percentage points (28.9%–40.1%), social impact by 7.9 (12.5%–20.4%), productivity impairment by 6.7 (10.9%–17.6%), mental health impact by 5.6 (10.8%–16.4%), and absenteeism by 4.2 (6.4%–10.6%). Above a score of 4 the trajectory diverged across domains: healthcare-seeking showed the most consistent monotonic increase, reaching 76.1% at a score of 10, while social and mental health impact showed steeper increments between scores of 5 and 8. Absenteeism, non-significant below a score of 4, rose steeply thereafter, reaching an unadjusted OR of 13.27 (95% CI: 7.58–23.21) at a score of 10.

## Discussion

### Main finding

In 11,529 desk-based working adults, a self-reported pain score of 4 was the lowest level at which the odds of functional impact were simultaneously and significantly elevated across four independent domains, productivity impairment, healthcare-seeking, mental health impact, and social participation, after adjustment for age and sex and after accounting for clustering of workers within employing organisations. Work absenteeism followed the same directional pattern with an association of similar magnitude but did not reach significance at a score of 4 once clustering was modelled, consistent with its rarity and its position as the most downstream consequence of pain. The finding of interest is therefore not that any single domain crosses a threshold, but that four conceptually distinct dimensions of the occupational impact of pain converge on the same relatively modest pain level.

This convergence is meaningful because the four domains capture different and largely independent aspects of working life: the capacity to work, the decision to seek care, psychological wellbeing, and participation in social and daily roles. That they become jointly elevated at a score of 4, rather than at the higher scores conventionally associated with severe pain, suggests that the functional cost of pain in working adults accumulates earlier than absence-based monitoring would detect. The anchor wording of the scale is consistent with this: a score of 4 marks the point at which pain shifts from something that can be ignored most of the time to something of which the person is constantly aware while still working through it.

### Interpreting the convergence point

It is important to be clear about what the convergence does and does not represent. Whether a given domain reaches statistical significance at a particular score level depends not only on the magnitude of the underlying association but also on the number of participants at that level and the base rate of the outcome. Absenteeism, the rarest outcome, was the last domain to reach significance, and its point estimate at a score of 3 was already above one (OR: 1.19) but did not reach significance. A score of 4 should therefore be understood as the earliest point of convergent statistical significance in this sample, influenced by domain base rates and statistical power, rather than as a biologically fixed inflection point. The close agreement between unadjusted and adjusted estimates, and the consistency of the convergence across all five domains, nonetheless support its practical interpretability as a screening signal.

### Contextualising against existing evidence

The designation of a score of 4 as the lower boundary of moderate pain has a long but inconsistently evidenced history (26, 27). Most of the literature establishing this boundary derives from single outcomes, commonly pain interference or analgesic response, in clinical populations (27, 28). The present findings add a multi-domain, population-level grounding for a score of 4 as a functionally meaningful point, derived from a working population in whom the consequences of pain span productivity, mental health, social life, and healthcare use simultaneously. A recent overview of cut-off points on the numerical rating scale found no consensus, with considerable variability across populations and outcomes (26). Our findings do not resolve that variability or imply a universal threshold; they show that, in this sample, a score of 4 is the lowest point of multi-domain functional significance, which has practical utility for workplace screening.

### Domain-specific patterns

Productivity impairment was the most prevalent consequence at moderate pain, consistent with evidence that presenteeism represents a greater economic burden than absenteeism. Productivity loss accumulates across the moderate range, well before pain drives formal absence, so systems focused mainly on absence miss the earlier and larger part of the burden. Absenteeism, by contrast, was the rarest domain, was concentrated within a subset of organisations, and did not reach significance at a score of 4 once clustering was accounted for; it became significant only at higher pain scores. This ordering, in which absence is the last and weakest domain to respond to rising pain, reinforces the central point: waiting for absence to signal need means intervening late, after productivity, mental health, healthcare, and social function have already been affected. A pain-intensity threshold for increased long-term sickness absence has been reported previously in an occupational cohort (23).

Healthcare-seeking was the earliest domain to reach significance and rose most consistently, reaching 76.1% at a score of 10; by a score of 4, 40.1% had already sought professional healthcare, a substantial demand signal at moderate intensity. Mental health and social impact rose steeply across the range, with marked escalation between scores of 5 and 8, consistent with the biopsychosocial model in which psychological and social consequences accumulate as pain exceeds the level at which it can be functionally absorbed (19). That these domains continued to escalate above a score of 4 rather than plateauing underlines the value of earlier review.

### Implications for occupational health practice

These findings carry implications for screening and earlier review. A single-item self-reported pain score, already embedded in many digital wellness platforms, can serve as a low-burden triage criterion. The evidence supports treating a score of 4 as a point at which proactive follow-up may be warranted, not because it is the most severe point on the scale, but because it is the lowest at which risk is simultaneously elevated and significant across the breadth of consequences relevant to working life. The implication is one of timing: review at a score of 4, through routes such as ergonomic assessment, earlier physiotherapy access, or workload adjustment, is consistent with a preventive rather than reactive model (29).

It is important to be precise about what this evidence does and does not support. These data show that a score of 4 is associated with significant and convergent elevation of functional burden across four independent domains, robust to age, sex, and organisational clustering. They do not show that intervening at a score of 4 improves outcomes. Establishing the clinical utility of a score of 4 as an intervention threshold, as distinct from a screening signal, requires prospective or experimental evidence beyond the scope of this study.

Because the pain score is already captured automatically by the digital assessment, a score-based flag could be embedded directly into platform workflows at negligible additional burden, prompting earlier review without requiring any new instrument. This is the practical appeal of a single-score signal in a digital occupational health setting, and it distinguishes the present approach from threshold work derived from clinical instruments that are not routinely collected at scale in the workplace.

### Strengths and limitations

This study has several strengths: a large real-world sample of desk-based workers, simultaneous analysis of five functional domains, a transparent maximum-score derivation that avoids double-counting, and confirmation that the convergence finding is robust to adjustment for age and sex.

Several limitations should be acknowledged. This was a cross-sectional study, so causal inferences cannot be drawn and the direction of associations cannot be established; for example, people with poorer mental health or reduced social participation may report higher pain independently of any causal pathway. Participants were self-selected working adults using an employer-sponsored platform, which may introduce selection bias, and workers with very high pain may be underrepresented if unable to complete the assessment or no longer in work. The sample came from a single UK platform, limiting generalisability. Functional outcomes were brief self-report items rather than validated multi-item instruments, and the productivity composite, although theoretically grounded, combines three related but distinct mechanisms. Pain intensity reflected each participant's maximum score across body regions, capturing peak rather than average or site-specific burden. Although we accounted for clustering of workers within organisations using cluster-robust standard errors and confirmatory GEE, residual confounding by unmeasured factors such as job demands, pain chronicity, and comorbidity remains possible. A further measurement limitation is that the impact question referred to current pain effects, whereas the absenteeism and healthcare-seeking questions asked whether the participant had ever taken time off or sought care, so these two outcomes use a lifetime rather than a current reference period; this temporal mismatch may attenuate or inflate their association with the current pain score and should be considered when interpreting those two domains.

A further consideration concerns the pain scale itself. The scale used defined verbal anchors of the descriptive pain scale type, displayed alongside the selected score; this anchoring is a strength in that it gave every respondent the same functional reference for each level, but it also means the descriptors have not been formally validated as a standalone instrument, and whether each respondent read the descriptor before selecting was not recorded. The convergence point should therefore be interpreted as specific to this anchored scale: a score of 4, defined here as constant awareness of pain while still able to continue most activities, may not be directly interchangeable with a score of 4 on an unanchored numerical rating scale used elsewhere. Encouragingly, the anchor wording gives the finding clear face validity: a score of 3 is defined as pain that “bothers me but I can ignore it most of the time,” whereas a score of 4 denotes being “constantly aware of my pain but can continue most activities,” so the 3-to-4 transition marks a shift from intermittent to constant awareness of pain that aligns with the point at which functional impact converged. This correspondence is supportive rather than confirmatory. The convergence point is also influenced by outcome base rates and statistical power and should not be read as a fixed biological inflection. Finally, although the analysis adjusted for age and sex and accounted for organisational clustering, residual confounding by unmeasured factors such as job demands, pain chronicity, or comorbidity remains possible; a dedicated analysis of sex differences is reserved for a separate planned study.

Several directions for future work follow from these limitations and from questions raised in review. First, the functional impact of pain plausibly varies by body region; a preliminary exploration by pain site in these data indicated that estimates were stable only for the highest-volume regions (lower back and neck) and that several sites had too few observations for reliable modelling, so a robust site-specific analysis would require dedicated, adequately powered methods and is an important next step. Second, thresholds may differ by occupational sector; industry could not be reliably determined in the present dataset and was therefore not analysed, but would be valuable where sector is captured directly. Third, and most importantly, prospective studies are needed to test whether review prompted at a score of 4 improves functional outcomes and reduces progression to work disability. A dedicated analysis of sex differences in the pain burden is also planned separately.

## Conclusion

Musculoskeletal pain is ubiquitous among desk-based workers, yet occupational health practice lacks an empirically grounded point at which to prompt earlier review. In 11,529 working adults assessed through a digital platform, a self-reported pain score of 4 was the lowest level at which the odds of functional impact were consistently and significantly elevated across four independent domains, productivity impairment, healthcare-seeking, mental health impact, and social participation, robust to age, sex, and clustering of workers within organisations. Work absenteeism followed the same direction but, as the rarest and most downstream outcome, did not reach significance at this level once clustering was modelled. Because the pain score is already captured automatically by the assessment, a score of 4 offers a low-burden, scalable signal that could be embedded in platform workflows to prompt earlier occupational health review, before the functional cost of pain has fully accumulated. These associations are cross-sectional; prospective studies are now needed to test whether acting at this threshold improves outcomes.

## Data Availability

The dataset analysed in this study is not publicly available. It comprises de-identified, individual-level records generated through routine use of a commercial workplace platform and is commercially sensitive. Enquiries about the data can be directed to the corresponding author and will be considered on reasonable request, subject to a data-sharing agreement and the approval of Vitrue Health.
