# Supplementary material for: Self-reported pain on a digital workplace assessment: identifying the score that flags desk-based workers for earlier occupational health review
Source: Front Digit Health. 2026 Aug 18;8:1919657. doi: 10.3389/fdgth.2026.1919657 (PMC13529524; doi:10.3389/fdgth.2026.1919657)
Supplement: Supplementary file 1 [file Supplementaryfile1.docx]

**Supplementary Material**

**Supplementary Table S1.**  Full verbal descriptors of the 11-point descriptive pain scale used in the digital assessment platform.

*Each numerical level is paired with a verbal anchor. Descriptors were displayed on hover and remained visible alongside the selected score. A score of 4 (highlighted) marks the shift from pain that can be ignored most of the time (3) to constant awareness of pain while still able to continue most activities (4). The descriptors are not a formally validated instrument.*

| **Score** | **Label** | **Verbal descriptor shown to respondent** |
| --- | --- | --- |
| 0 | Pain free | I have no pain, my life is completely unaffected |
| 1 | Very mild | My pain is hardly noticeable |
| 2 | Discomforting | I have a low level of pain. I am aware of my pain only when I pay attention to it |
| 3 | Tolerable | My pain bothers me but I can ignore it most of the time |
| **4** | **Distressing** | **I am constantly aware of my pain but can continue most activities** |
| 5 | Very distressing | I think about my pain most of the time. I cannot do some activities because of it |
| 6 | Intense | I think about my pain all of the time. I give up many activities because of it |
| 7 | Very intense | I am in pain all of the time. It keeps me from doing most activities |
| 8 | Utterly horrible | My pain is so severe it is hard to think of anything else. Talking and listening are difficult |
| 9 | Unbearable | My pain is all I can think about. I can barely move or talk because of the pain |
| 10 | Unimaginable | I am in bed and can't move because of the pain. I need to go to the hospital |

**Supplementary Table S2.**  Multi-select pain-impact question and mapping of each response option to the functional domains.

*Verbatim question: “What impact has your pain had on you?” (optional; respondents could select any number of options). Three options formed the productivity composite; one each formed the mental health and social domains; “Other” was not analysed; and “my pain doesn't really impact me” corresponded to no impact domain endorsed.*

| **Response option (verbatim)** | **Mapped domain** |
| --- | --- |
| Anxiety, stress or mood changes | Mental health impact |
| Reduced work productivity | Productivity impairment |
| Sleep disruption | Productivity impairment |
| Difficulty concentrating | Productivity impairment |
| Interference with social life and daily tasks | Social participation (social impact) |
| Other | Not mapped (not analysed) |
| My pain doesn't really impact me | No domain endorsed (all impact domains = 0) |

**Supplementary Table S3.**  Confirmatory generalised estimating equation (GEE) models: age- and sex-adjusted odds ratios (95% CI) by pain score, exchangeable working correlation, organisation as grouping variable.

| **NRS** | **Productivity** | **Healthcare-seeking** | **Mental health** | **Social** | **Absenteeism** |
| --- | --- | --- | --- | --- | --- |
| 2 | 1.09 (0.63-1.86) | 1.35 (0.82-2.24) | 1.09 (0.70-1.71) | 1.27 (0.83-1.94) | 0.73 (0.37-1.45) |
| 3 | 2.09 (1.24-3.52)** | 2.14 (1.23-3.71)** | 2.32 (1.36-3.94)** | 2.81 (1.74-4.54)*** | 1.25 (0.56-2.77) |
| **4** | **3.43 (1.92-6.10)***** | **3.62 (1.96-6.69)***** | **3.63 (2.30-5.73)***** | **4.80 (3.06-7.53)***** | **2.13 (0.84-5.40)** |
| 5 | 4.51 (2.28-8.92)*** | 5.49 (3.41-8.85)*** | 6.58 (4.06-10.67)*** | 10.64 (7.35-15.41)*** | 3.70 (1.51-9.08)** |
| 6 | 6.78 (3.74-12.28)*** | 6.26 (3.92-10.00)*** | 8.54 (5.02-14.54)*** | 11.47 (8.02-16.40)*** | 4.39 (2.01-9.57)*** |
| 7 | 9.82 (5.60-17.23)*** | 9.19 (6.32-13.37)*** | 11.72 (7.08-19.41)*** | 16.45 (11.27-24.01)*** | 6.03 (3.15-11.55)*** |
| 8 | 12.23 (6.91-21.65)*** | 10.92 (7.38-16.14)*** | 17.74 (11.02-28.58)*** | 18.82 (12.27-28.85)*** | 8.66 (4.04-18.54)*** |
| 9 | 16.57 (11.45-23.98)*** | 12.74 (8.01-20.26)*** | 22.10 (11.62-42.06)*** | 24.26 (16.23-36.24)*** | 9.90 (6.38-15.35)*** |
| 10 | 22.32 (13.72-36.31)*** | 16.50 (9.94-27.40)*** | 29.87 (18.45-48.37)*** | 35.93 (21.10-61.17)*** | 11.94 (5.76-24.73)*** |

*Values are odds ratios (95% CI). * p<0.05; ** p<0.01; *** p<0.001. GEE estimates are materially consistent with the primary cluster-robust models in Table 2, including the attenuation of absenteeism at a score of 4.*
